# Suppression of *cdc13-2*-associated senescence by *pif1-m2* requires Ku-mediated telomerase recruitment

**DOI:** 10.1093/g3journal/jkab360

**Published:** 2021-10-13

**Authors:** Enikő Fekete-Szücs, Fernando R Rosas Bringas, Sonia Stinus, Michael Chang

**Affiliations:** European Research Institute for the Biology of Ageing, University of Groningen, University Medical Center Groningen, Groningen 9713 AV, The Netherlands

**Keywords:** Cdc13, Pif1, Ku complex, replicative senescence, telomerase recruitment

## Abstract

In *Saccharomyces cerevisiae*, recruitment of telomerase to telomeres requires an interaction between Cdc13, which binds single-stranded telomeric DNA, and the Est1 subunit of telomerase. A second pathway involving an interaction between the yKu complex and telomerase RNA (TLC1) contributes to telomerase recruitment but cannot sufficiently recruit telomerase on its own to prevent replicative senescence when the primary Cdc13-Est1 pathway is abolished—for example, in the *cdc13-2* mutant. In this study, we find that mutation of *PIF1*, which encodes a helicase that inhibits telomerase, suppresses the replicative senescence of *cdc13-2* by increasing reliance on the yKu-TLC1 pathway for telomerase recruitment. Our findings reveal new insight into telomerase-mediated telomere maintenance.

## Introduction

Telomeres are composed of G/C-rich repetitive sequences at the termini of eukaryotic chromosomes and play a pivotal role in genome maintenance by “capping” chromosome ends, preventing them from unwanted nucleolytic degradation, homologous recombination, and fusion with neighboring chromosomes (Jain and Cooper 2010). In addition, to overcome progressive telomere shortening due to the end replication problem, telomeres are elongated by a specialized reverse transcriptase called telomerase. In the budding yeast *Saccharomyces cerevisiae*, telomerase is minimally composed of the protein subunit Est2 and the RNA subunit TLC1 (Singer and Gottschling 1994; Lingner *et al.* 1997). However, additional accessory proteins, Est1 and Est3, are required for telomerase activity *in vivo* and are thought to be involved in the recruitment and/or activation of telomerase (Wellinger and Zakian 2012). Eliminating any of the Est proteins or TLC1 results in an “ever shorter telomeres” (*est*) phenotype characterized by progressive telomere shortening that ultimately leads to replicative senescence (Lundblad and Szostak 1989; Singer and Gottschling 1994; Lendvay *et al.* 1996).

Maintaining telomere length homeostasis through the regulation of telomerase is essential for genome stability. Several lines of evidence suggest that the recruitment of telomerase to telomeres involves a direct interaction between the Est1 subunit of telomerase and Cdc13, a protein that binds single-strand telomeric DNA with high affinity (Lin and Zakian 1996; Nugent *et al.* 1996). Expression of a Cdc13-Est2 fusion protein can support telomere maintenance in an *est1Δ* null mutant, suggesting that the main function of Est1 is to bring telomerase to telomeres (Evans and Lundblad 1999). Cdc13 is essential for telomere capping, so a null mutation is lethal; however, an extensively studied point mutant, *cdc13-2*, is not capping defective but displays an *est* phenotype (Nugent *et al.* 1996). The amino acid mutated in *cdc13-2*, E252, lies within the recruitment domain (RD), which is able to recruit telomerase to telomeres when fused to the DNA-binding domain of Cdc13 (Pennock *et al.* 2001). The mutation (E252K) results in a charge swap and can be suppressed by *est1-60*, which encodes a mutant Est1 with a reciprocal charge swap (K444E), suggesting a direct physical interaction between the two proteins (Pennock *et al.* 2001). Consistent with this idea, purified full-length Cdc13 and Est1 interact *in vitro* (Wu and Zakian 2011), and structural analysis revealed two conserved motifs within the Cdc13 RD, called Cdc13_EBM-N_ and Cdc13_EBM-C_ (referring to N- and C-terminal Est1-binding motifs, respectively), responsible for this interaction (Chen *et al.* 2018). The Cdc13 E252K mutation resides within the latter motif. Surprisingly, mutations in the Cdc13_EBM-C_ motif, including E252K, do not abolish the interaction between Cdc13 and Est1 *in vitro* despite causing a dramatic reduction in Est1 telomere association *in vivo* (Chan *et al.* 2008; Wu and Zakian 2011; Chen *et al.* 2018). Thus, the mechanism by which the Cdc13_EBM-C_ motif promotes telomerase-mediated telomere extension is still unclear.

In contrast, mutations in Cdc13_EBM-N_ abolish the Cdc13-Est1 interaction *in vitro*, yet only result in a modest reduction in Est1 telomere association and short, but stable, telomere length *in vivo* (Chen *et al.* 2018). This telomerase recruitment pathway works in parallel with a second pathway involving Sir4, the yKu complex, and TLC1. Double-strand telomeric DNA is bound by Rap1 (Buchman *et al.* 1988; Conrad *et al.* 1990), which interacts with Sir4 (Moretti *et al.* 1994). Sir4, in turn, interacts with the Yku80 subunit of the yKu complex (Roy *et al.* 2004), which binds to the tip of a 48-nt hairpin in TLC1 (Peterson *et al.* 2001; Stellwagen *et al.* 2003; Chen *et al.* 2018). Mutations that abolish the yKu-TLC1 interaction (*e.g., tlc1*Δ*48* and *yku80-135i*) result in slightly short but stable telomeres (Peterson *et al.* 2001; Stellwagen *et al.* 2003), much like Cdc13_EBM-N_ mutations. Disrupting both the yKu-TLC1 interaction and Cdc13_EBM-N_-Est1 interaction results in an *est* phenotype (Chen *et al.* 2018).

Pif1, a 5′–3′ helicase that is evolutionary conserved from bacteria to humans, directly inhibits telomerase activity at telomeres and DNA double-strand breaks (Schulz and Zakian 1994). Pif1 has both mitochondrial and nuclear isoforms; by altering the first (*pif1-m1*) and the second (*pif1-m2*) translational start sites, the functions can be separated (Schulz and Zakian 1994). The *pif1-m2* mutant abolishes nuclear Pif1 and, similar to *pif1Δ*, has elongated telomeres (Schulz and Zakian 1994). *In vitro*, purified Pif1 reduces telomerase processivity and displaces telomerase from telomeric oligonucleotides (Boulé *et al.* 2005). *In vivo*, deletion of *PIF1* increases telomere association of Est1, while overexpression of *PIF1* reduces telomere association of Est1 and Est2 (Boulé *et al.* 2005).

We previously showed that a double-strand break adjacent to at least 34 bp of telomeric sequence is efficiently extended by telomerase, resulting in the addition of a *de novo* telomere, but this does not occur in Cdc13_EBM-C_ mutants, such as *cdc13-2* (Strecker *et al.* 2017). Surprisingly, we found that the lack of telomere addition in *cdc13-2* cells can be suppressed by the *pif1-m2* mutation (Strecker *et al.* 2017). In this study, we find that *pif1-m2* suppresses the replicative senescence caused by the *cdc13-2* mutation in a manner dependent on the yKu-TLC1 telomerase recruitment pathway. In addition, *pif1-m2* suppresses the replicative senescence caused by disrupting both the yKu-TLC1 and Cdc13_EBM-N_–Est1 interactions. These observations provide new insight into the complexity of telomerase-mediated telomere maintenance.

## Materials and methods

### Yeast strains and plasmids

All yeast strains used in this study are listed in Table  1. Standard yeast genetic and molecular methods were used (Sherman 2002; Amberg *et al.* 2005). The YEp24-*CDC13* plasmid was first described in an article from the Hartwell lab, where it was originally designated YEp24-*CDC13*-161-4 (Garvik *et al.* 1995). Plasmids pEFS4 (pRS415-*cdc13-F237A*) and pFR96 (pRS415-*cdc13-F237A*, *E252K*) were created by site-directed mutagenesis of pDD4317 (pRS415-CDC13; Strecker *et al.* 2017) using primers designed by NEBaseChanger and the Q5 Site-Directed Mutagenesis Kit (New England Biolabs, Cat. No.: E0554S). The mutations were confirmed by DNA sequencing.

**Table 1:** Yeast strains used in this study

Strain name	Genotype	Source
DDY3768	*MAT* **a** *-inc ura3-52 lys2-801 ade2-101 trp1-Δ63 his3-Δ200 leu2::natMX rad52::HIS3 VII-L::TG34-HOcs-LYS2 ura3::hphMX cdc13::kanMX* YEp24-*CDC13* pRS425-*CDC13*	Strecker et al. (2017)
DDY3778	*MAT* **a** *-inc ura3-52 lys2-801 ade2-101 trp1-Δ63 his3-Δ200 leu2::natMX rad52::HIS3 VII-L::TG34-HOcs-LYS2 ura3::hphMX cdc13::kanMX* YEp24-*CDC13* pRS425-*cdc13-E252K*	Strecker et al. (2017)
DDY3783	*MAT* **a** *-inc ura3-52 lys2-801 ade2-101 trp1-Δ63 his3-Δ200 leu2::natMX rad52::HIS3 VII-L::TG34-HOcs-LYS2 ura3::hphMX cdc13::kanMX pif1-m2* YEp24-*CDC13* pRS425-*CDC13*	Strecker et al. (2017)
DDY3793	*MAT* **a** *-inc ura3-52 lys2-801 ade2-101 trp1-Δ63 his3-Δ200 leu2::natMX rad52::HIS3 VII-L::TG34-HOcs-LYS2 ura3::hphMX cdc13::kanMX pif1-m2* YEp24-*CDC13* pRS425-*cdc13-E252K*	Strecker et al. (2017)
VSY20	*MAT* **a** */MAT*α *ade2-1/ADE2 can1-100/can1-100 his3-11,15/his3-11,15 leu2-3,112/leu2-3,112 trp1-1/trp1-1 ura3-1/ura3-1 RAD5/RAD5 cdc13-2::natMX/CDC13 pif1-m2/PIF1 yku80-135i::kanMX/YKU80*	This study
VSY7	*MAT* **a** */MAT*α *ade2-1/ADE2 can1-100/can1-100 his3-11,15/his3-11,15 leu2-3,112/leu2-3,112 trp1-1/trp1-1 ura3-1/ura3-1 RAD5/RAD5 cdc13-2::natMX/CDC13 pif1-m2/PIF1 tlc1Δ48::kanMX/TLC1*	This study
EFSY142	*MAT* **a** */MAT*α *ade2-1/ADE2 can1-100/can1-100 his3-11,15/his3-11,15 leu2-3,112/leu2-3,112 trp1-1/trp1-1 ura3-1/ura3-1 RAD5/RAD5 cdc13-2::natMX/CDC13 pif1-m2/PIF1 sir4ΔhphMX/TLC1*	This study
EFSY73	*MAT* **a** */MAT*α *ADE2/ADE2 can1-100/can1-100 his3-11,15/his3-11,15 leu2-3,112/leu2-3,112 trp1-1/trp1-1 ura3-1/ura3-1 RAD5/RAD5 cdc13::kanMX/CDC13 pif1-m2/PIF1 tlc1Δ48::hphMX/TLC1* pRS415-*cdc13-F237A*	This study
FRY867	*MAT* **a** */MAT*α *ade2-1/ade2-1 can1-100/can1-100 his3-11,15/his3-11,15 leu2-3,112/leu2-3,112 trp1-1/trp1-1 ura3-1/ura3-1 RAD5/RAD5 cdc13::kanMX/CDC13 pif1-m2/PIF1 tlc1Δ48::hphMX/TLC1* pRS415-*cdc13-F237A/E252K*	This study
CAY2	*MAT* **a** */MAT*α *ade2-1/ADE2 can1-100/can1-100 his3-11,15/his3-11,15 leu2-3,112/leu2-3,112 trp1-1/trp1-1 ura3-1/ura3-1 RAD5/RAD5* *est1::HIS3/EST1 pif1-m2/PIF1*	This study
MCY815	*MAT* **a** */MAT*α *ADE2/ADE2 can1-100/can1-100 his3-11,15/his3-11,15 leu2-3,112/leu2-3,112 trp1-1/trp1-1 ura3-1/ura3-1 RAD5/RAD5 mec1-21/MEC1 tel1ΔURA3/TEL1 pif1-m2/PIF1*	This study
EFSY8	*MAT* **a** */MAT*α *ade2-1/ADE2 can1-100/can1-100 his3-11,15/his3-11,15 leu2-3,112/leu2-3,112 trp1-1/trp1-1 ura3-1/ura3-1 RAD5/RAD5 cdc13-2::natMX/CDC13 pif1-m2/PIF1 rif1ΔHIS3MX/RIF1*	This study
EFSY9	*MAT* **a** */MAT*α *ade2-1/ADE2 can1-100/can1-100 his3-11,15/his3-11,15 leu2-3,112/leu2-3,112 trp1-1/trp1-1 ura3-1/ura3-1 RAD5/RAD5 cdc13-2::natMX/CDC13 pif1-m2/PIF1 rif2ΔHIS3MX/RIF2*	This study
EFSY31	*MAT* **a** */MAT*α *ade2-1/ADE2 can1-100/can1-100 his3-11,15/his3-11,15 leu2-3,112/leu2-3,112 trp1-1/trp1-1 ura3-1/ura3-1 RAD5/RAD5 cdc13-2::natMX/CDC13 pif1-m2/PIF1 tel1ΔURA3/TEL1*	This study

### Liquid culture senescence assay

Liquid culture senescence assays were performed essentially as previously described (van Mourik *et al.* 2016). Each senescence assay started with diploid strains. Freshly dissected haploid spores were allowed to form colonies on YPD agar plates after two days of growth at 30°C. Cells from these colonies were serially passaged in liquid culture medium at 24-h intervals. For each passage, the cell density of each culture was measured by optical density (calibrated by cell counting using a haemocytometer), and the cultures were diluted back into fresh medium at a cell density of 2 × 10^5^ cells/ml. Cell density was plotted as a function of population doublings.

### Telomere Southern blot

Telomere length analysis by Southern blotting was performed essentially as previously described (van Mourik *et al.* 2018). A 1.8-kb DNA fragment containing telomeric sequences generated from the BsmAI-digestion of plasmid pYt103 (Shampay *et al.* 1984) was loaded together with each sample. Southern blots were probed with a telomere-specific probe (5′-TGTGGGTGTGGTGTGTGGGTGTGGTG-3′).

## Results and discussion

### Mutation of *PIF1* suppresses the replicative senescence caused by the *cdc13-2* mutation

**Figure 1:**
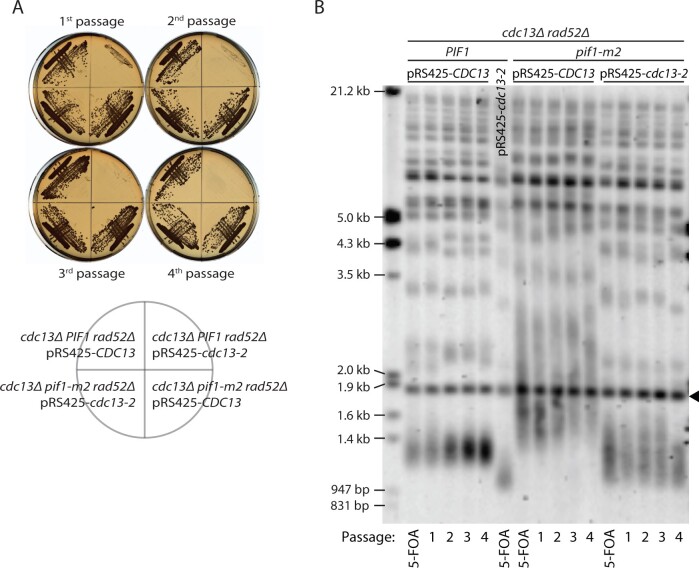
*cdc13-2 pif1-m2* cells do not senesce and have telomeres that are stable in length. (A) Strains of the indicated genotypes were passaged four times on YPD plates after counter selection on 5-FOA to remove plasmid YEp24*-CDC13*. Each passage corresponds to ∼25 generations of growth. (B) Telomere Southern blot analysis of strains from A. The black arrowhead indicates a 1.8-kb DNA fragment generated from the BsmAI-digestion of plasmid pYt103 loaded as a control.

To investigate how telomere addition is possible in a *cdc13-2 pif1-m2* genetic background, we serially passaged cells to determine whether they would senesce. For these experiments, we used strains from our previous study (Strecker *et al.* 2017): *cdc13Δ* or *cdc13Δ pif1-m2* cells, kept alive by the presence of a high-copy plasmid expressing wild-type *CDC13* and the *URA3* gene, transformed with an additional high-copy plasmid containing either *CDC13* or *cdc13-2*. These cells also carried a deletion of *RAD52* to prevent homologous recombination-mediated telomere maintenance (Claussin and Chang 2015). We then counterselected the first plasmid by growing cells on media containing 5-fluoroorotic acid (5-FOA), which is toxic to cells expressing *URA3*. 5-FOA-resistant colonies were subsequently serially passaged on agar plates (Figure  1A). Senescence was apparent for *cdc13-2 PIF1* cells already after the first passage, whereas *CDC13* and *cdc13-2 pif1-m2* strains did not show any sign of senescence even after the fourth passage. We analyzed the telomere length of these strains and found that, consistent with previous studies, *pif1-m2* has increased telomere length compared with wild type (Schulz and Zakian 1994) while the telomeres are very short in the *cdc13-2* mutant (Lendvay *et al.* 1996; Nugent *et al.* 1996). Interestingly, *cdc13-2 pif1-m2* telomeres are approximately wild-type in length, albeit more heterogeneous, and stable throughout the course of the experiment (Figure  1B). Our findings indicate that telomerase-mediated telomere extension can occur in *cdc13-2 pif1-m2* cells, allowing cells to maintain telomere length homeostasis and avoid replicative senescence. Because the strains used for this experiment have an unusual genotype (Table  1), relevant for our previous study (Strecker *et al.* 2017) but not for this study, we performed all subsequent experiments in a different strain background (W303), where none of the genes were overexpressed.

### The yKu-TLC1 telomerase recruitment pathway is necessary to maintain telomere length in *cdc13-2 pif1-m2* cells

**Figure 2:**
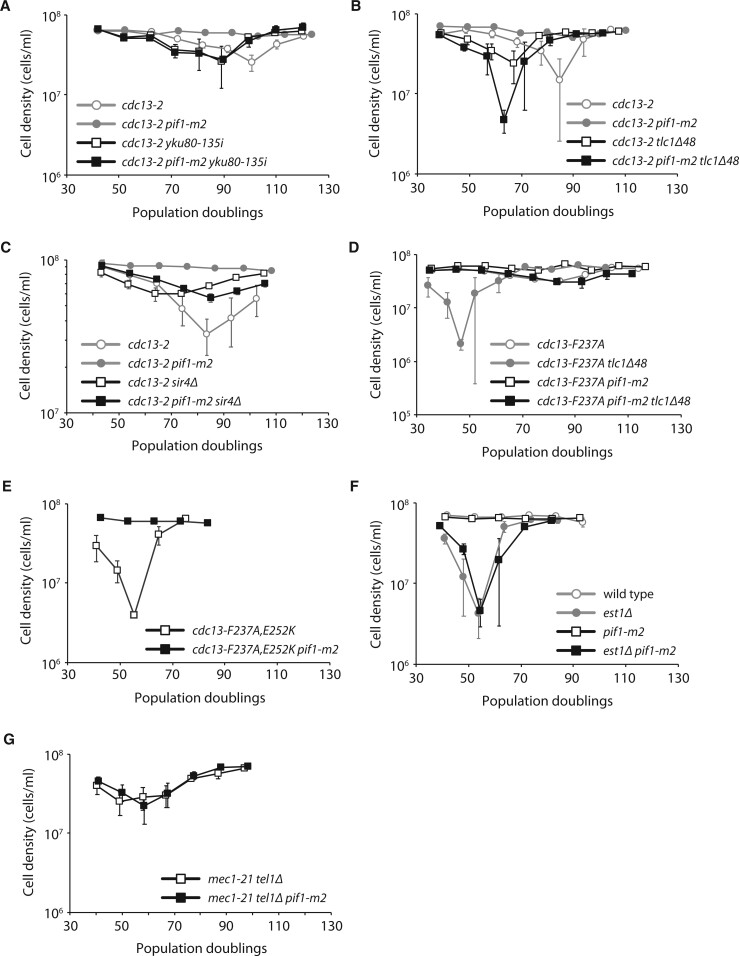
Telomeres are maintained by the yKu-TLC1 pathway in *cdc13-2 pif1-m2* cells. Senescence was monitored in liquid culture by serial passaging of haploid meiotic progeny derived from the sporulation of VSY20 (A), VSY7 (B), EFSY142 (C), EFSY73 (D), FRY867 (E), CAY2 (F), and MCY815 (G). Average cell density ±SEM of 3–9 independent isolates per genotype (except *n* = 2 for *cdc13-F237A, E252K*) is plotted.

We hypothesized that the yKu-TLC1 pathway may become essential for telomere length homeostasis in *cdc13-2 pif1-m2* strains. To test this possibility, haploid meiotic progeny derived from the sporulation of *CDC13/cdc13-2 PIF1/pif1-m2 YKU80/yku80-135i* and *CDC13/cdc13-2 PIF1/pif1-m2 TLC1/tlc1Δ48* heterozygous diploids were serially propagated in liquid culture for several days (Figure  2, A and B). The *yku80-135i* and *tlc1Δ48* alleles disrupt the interaction between the yKu complex and TLC1 (Peterson *et al.* 2001; Stellwagen *et al.* 2003). As expected, *cdc13-2* cultures grew slower as the experiment progressed and cells senesced, but growth was eventually restored upon the emergence of survivors that utilize recombination-mediated mechanisms to maintain telomeres (Lendvay *et al.* 1996). In contrast, the *cdc13-2 pif1-m2* strains did not senesce, confirming our previous observations in a different strain background (S288C for strains used in Figure  1 as opposed to W303 for all other strains used in this study). The *cdc13-2 pif1-m2 yku80-135i* and *cdc13-2 pif1-m2 tlc1Δ48* triple mutants showed a pattern of senescence and survivor formation, indicating that the yKu-TLC1 telomerase recruitment pathway is required for telomere length homeostasis in *cdc13-2 pif1-m2* cells. The *yku80-135i* and *tlc1Δ48Δ* alleles caused *cdc13-2* and *cdc13-2 pif1-m2* strains to senesce faster, but the reason for this is currently unclear.

The abundance of TLC1 RNA is reduced to ∼30% and ∼48% in *yku80-135i* and *tlc1Δ48* mutants, respectively, compared with wild-type cells (Zappulla *et al.* 2011). In addition, disrupting the yKu-TLC1 interaction causes mislocalization of TLC1 to the cytoplasm (Gallardo *et al.* 2008; Pfingsten *et al.* 2012). It is possible that reduced abundance and/or mislocalization of TLC1, rather than disruption of the yKu-TLC1 telomerase recruitment pathway, is responsible for the senescence of *cdc13-2 pif1-m2 yku80-135i* and *cdc13-2 pif1-m2 tlc1Δ48* triple mutants. Sir4 is also required for the yKu-TLC1 recruitment pathway, but deletion of *SIR4* does not affect TLC1 abundance (Hass and Zappulla 2015), and there is no evidence that *sir4Δ* affects TLC1 localization. We find that *cdc13-2 pif1-m2 sir4Δ* triple mutants also senesce (Figure  2C), although the “dip” in the senescence curve was more shallow (note the difference in scale on the *y*-axis). The shallow dip is consistent across multiple isolates of *cdc13-2 pif1-m2 sir4Δ* (nine isolates) as well as *cdc13-2 sir4Δ* (six isolates). The presence of the shallow dip in *cdc13-2 sir4Δ* indicates that this effect is due to *sir4Δ*, and is unrelated to the *pif1-m2* suppression of *cdc13-2* senescence. This effect of *sir4Δ* has also been observed with respect to the senescence of *mre11Δ yku80Δ* double mutants, which was attributed to increased recombination and amplification of Y′ subtelomeric elements (Liu *et al.* 2021). While these experiments leave open the possibility that reduced abundance and/or mislocalization of TLC1 plays a role in the senescence of *cdc13-2 pif1-m2* cells with an additional *yku80-135i*, *tlc1Δ48*, or *sir4Δ* mutation, the simplest interpretation of our findings is that recruitment of telomerase via the yKu-TLC1 pathway is indeed required for telomere length homeostasis in *cdc13-2 pif1-m2* cells.

Combining mutations that disrupt the Cdc13_EBM-N_–Est1 interaction (*e.g.*, *cdc13-F237A*) and the yKu-TLC1 interaction leads to replicative senescence (Chen *et al.* 2018). We tested whether the *pif1-m2* mutation could suppress this replicative senescence and found that it can: *cdc13-F237A tlc1Δ48* strains senesce while *cdc13-F237A pif1-m2 tlc1Δ48* strains do not (Figure  2D). Similarly, *pif1-m2* can suppress replicative senescence of a *cdc13-F237A, E252K* mutant that disrupts both the Cdc13_EBM-N_ and Cdc13_EBM-C_ motifs (Figure  2E).

In summary, these findings indicate that mutation of *PIF1* allows sufficient telomerase recruitment to avoid replicative senescence caused by disruption of the Cdc13_EBM-C_–Est1 interaction alone, or double disruption of both the Cdc13_EBM-N_–Est1 and yKu–TLC1 interactions. However, suppression is not possible when both the Cdc13_EBM-C_–Est1 and yKu–TLC1 interactions are abolished. Disruption of both the Cdc13_EBM-N_–Est1 and Cdc13_EBM-C_–Est1 interactions can be suppressed by mutation of *PIF1* (Figure  2E), suggesting that the Cdc13_EBM-N_–Est1 interaction plays a more minor role, likely in support of the Cdc13_EBM-C_–Est1 interaction. Our findings suggest that Pif1 inhibits telomerase regardless of how telomerase is recruited: mutation of *PIF1* in *cdc13-2* cells allows increased telomerase recruitment via the yKu-TLC1 pathway, while mutation of *PIF1* in *cdc13-F237A tlc1Δ48* cells allows increased telomerase recruitment via the Cdc13_EBM-C_-Est1 pathway.

### Mutation of *PIF1* cannot suppress the replicative senescence of *est1Δ*

The *cdc13-2* mutation greatly reduces the recruitment of Est1 to telomeres (Chan *et al.* 2008), and the expression of Cdc13-Est2 fusion protein allows cells to stably maintain their telomeres in the absence of Est1 (Evans and Lundblad 1999). Therefore, it was possible that *pif1-m2* suppresses the replicative senescence caused by *cdc13-2* by somehow bypassing the need for Est1 for telomerase-mediated telomere extension. To test this idea, we sporulated an *EST1/est1Δ PIF1/pif1-m2* heterozygous diploid and monitored growth of the haploid meiotic progeny (Figure  2F). We find that *est1Δ pif1-m2* double mutants senesce like *est1Δ* single mutants, indicating that mutation of *PIF1* cannot bypass the need for Est1.

### Tel1 acts through the Cdc13_EBM-C_ motif to regulate telomere length

**Figure 3:**
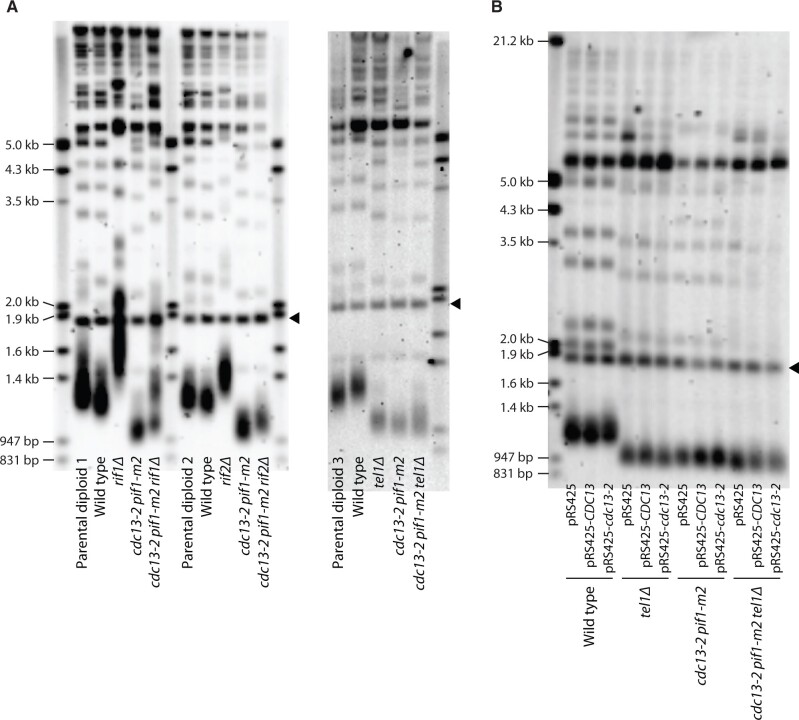
The *cdc13-2* allele is epistatic to *rif2Δ* and *tel1Δ* with respect to telomere length regulation in a *pif1-m2* background. Telomere Southern blot analysis of strains of the indicated genotypes. All strains were propagated for at least 100 population doublings before Southern blot analysis. Parental diploids 1, 2, and 3 are EFSY8, EFSY9, and EFSY31, respectively. The black arrowhead indicates a 1.8-kb DNA fragment generated from the BsmAI-digestion of plasmid pYt103 loaded as a control.

Because the *cdc13-2* mutation normally results in a complete defect in telomerase-mediated telomere extension, it has not been possible to perform classical genetic epistasis experiments to determine which telomere length regulators act through the Cdc13_EBM-C_-Est1 pathway. The viability and non-senescence of *cdc13-2 pif1-m2* strains give us the opportunity to do so. The Rap1-interacting factors, Rif1 and Rif2, negatively regulate telomerase (Hardy *et al.* 1992; Wotton and Shore 1997) while the Tel1 kinase is a positive regulator (Greenwell *et al.* 1995). We measured the telomere length of haploid strains propagated for over 100 population doublings after being generated from the sporulation of heterozygous diploids (Figure  3A). We find that *cdc13-2 pif1-m2* cells have short telomeres, which is in contrast to the more wild-type, but heterogeneous, length telomeres shown in Figure  1. The difference is most likely due to different genetic backgrounds (strains in Figure  1 are of the S288C background, with an additional deletion of *RAD52*, while all other strains used in this study are of the W303 background; Table  1), but not due to *cdc13-2* being expressed from a high-copy plasmid in Figure  1, because overexpression of neither *CDC13* nor *cdc13-2* affects telomere length (Figure  3B). While deletion of *RIF1* elongates *cdc13-2 pif1-m2* telomeres, both *cdc13-2 pif1-m2 rif2Δ* and *cdc13-2 pif1-m2 tel1Δ* triple mutants have very similar telomere lengths compared with *cdc13-2 pif1-m2*, indicating that Rif2 and Tel1 function upstream and in the same pathway as the Cdc13_EBM-C_ motif (Figure  3). Our results are consistent with previous observations showing that *tel1Δ* is epistatic to *rif2Δ* in terms of telomere length, while the relationship between Tel1 and Rif1 is more complex and telomere-specific (Craven and Petes 1999; Sholes *et al.* 2021).

Tel1 often functions in concert with a related kinase, Mec1. Mutation of both *MEC1* and *TEL1* results in an *est* phenotype (Ritchie *et al.* 1999). Because Tel1 promotes telomerase activity through the Cdc13_EBM-C_–Est1 interaction, we examined whether the same is true for Mec1. If so, the replicative senescence of *mec1 tel1* double mutants, like *cdc13-2*, should also be suppressed by *pif1-m2*. We sporulated a *MEC1/mec1-21 TEL1/tel1Δ PIF1/pif1-m2* diploid strain and monitored the growth of the *mec1-21 tel1Δ* and *mec1-21 tel1Δ pif1-m2* haploid meiotic progeny. Both strains exhibited a similar rate of senescence (Figure  2G), indicating that *pif1-m2* cannot suppress the *est* phenotype of a *mec1 tel1* double mutant, and that Mec1 functions in a different pathway than Tel1 to promote telomerase activity, as previously proposed (Ritchie *et al.* 1999; Keener *et al.* 2019).

In summary, our findings provide new insight into how telomerase is recruited to telomeres in *S. cerevisiae*. Further work is needed to determine how the Cdc13_EBM-C_ motif functions, what its relationship is with the Cdc13_EBM-N_ motif, and the role of Tel1 in promoting telomerase recruitment.

## Data availability

Strains and plasmids are available upon request. The authors affirm that all data necessary for confirming the conclusions of the article are present within the article, figures, and tables.

## References

[jkab360-B1] Amberg DC , Burke D, Strathern JN, Burke D; Cold Spring Harbor Laboratory. 2005. Methods in Yeast Genetics: A Cold Spring Harbor Laboratory Course Manual. Cold Spring Harbor, NY: Cold Spring Harbor Laboratory Press.

[jkab360-B2] Boulé JB , Vega LR, Zakian VA. 2005. The yeast Pif1p helicase removes telomerase from telomeric DNA. Nature. 438:57–61. 16121131 10.1038/nature04091

[jkab360-B3] Buchman AR , Kimmerly WJ, Rine J, Kornberg RD. 1988. Two DNA-binding factors recognize specific sequences at silencers, upstream activating sequences, autonomously replicating sequences, and telomeres in *Saccharomyces cerevisiae*. Mol Cell Biol. 8:210–225. 3275867 10.1128/mcb.8.1.210-225.1988 PMC363104

[jkab360-B4] Chan A , Boulé JB, Zakian VA. 2008. Two pathways recruit telomerase to *Saccharomyces cerevisiae* telomeres. PLoS Genet. 4:e1000236. 18949040 10.1371/journal.pgen.1000236 PMC2567097

[jkab360-B5] Chen H , Xue J, Churikov D, Hass EP, Shi S, et al 2018. Structural insights into yeast telomerase recruitment to telomeres. Cell. 172:331–343 e313. 29290466 10.1016/j.cell.2017.12.008 PMC5839504

[jkab360-B6] Claussin C , Chang M. 2015. The many facets of homologous recombination at telomeres. Microb Cell. 2:308–321. 28357308 10.15698/mic2015.09.224 PMC5354574

[jkab360-B7] Conrad MN , Wright JH, Wolf AJ, Zakian VA. 1990. RAP1 protein interacts with yeast telomeres in vivo: overproduction alters telomere structure and decreases chromosome stability. Cell. 63:739–750. 2225074 10.1016/0092-8674(90)90140-a

[jkab360-B8] Craven RJ , Petes TD. 1999. Dependence of the regulation of telomere length on the type of subtelomeric repeat in the yeast *Saccharomyces cerevisiae*. Genetics. 152:1531–1541. 10430581 10.1093/genetics/152.4.1531 PMC1460705

[jkab360-B9] Evans SK , Lundblad V. 1999. Est1 and Cdc13 as comediators of telomerase access. Science. 286:117–120. 10506558 10.1126/science.286.5437.117

[jkab360-B10] Gallardo F , Olivier C, Dandjinou AT, Wellinger RJ, Chartrand P. 2008. *TLC1* RNA nucleo-cytoplasmic trafficking links telomerase biogenesis to its recruitment to telomeres. Embo J. 27:748–757. 18273059 10.1038/emboj.2008.21 PMC2265757

[jkab360-B11] Garvik B , Carson M, Hartwell L. 1995. Single-stranded DNA arising at telomeres in *cdc13* mutants may constitute a specific signal for the *RAD9* checkpoint. Mol Cell Biol. 15:6128–6138. 7565765 10.1128/mcb.15.11.6128 PMC230864

[jkab360-B12] Greenwell PW , Kronmal SL, Porter SE, Gassenhuber J, Obermaier B, et al 1995. *TEL1*, a gene involved in controlling telomere length in *S. cerevisiae*, is homologous to the human ataxia telangiectasia gene. Cell. 82:823–829. 7671310 10.1016/0092-8674(95)90479-4

[jkab360-B13] Hardy CF , Sussel L, Shore D. 1992. A RAP1-interacting protein involved in transcriptional silencing and telomere length regulation. Genes Dev. 6:801–814. 1577274 10.1101/gad.6.5.801

[jkab360-B14] Hass EP , Zappulla DC. 2015. The Ku subunit of telomerase binds Sir4 to recruit telomerase to lengthen telomeres in *S. cerevisiae*. eLife. 4:10.7554/eLife.07750 PMC4547093 26218225

[jkab360-B15] Jain D , Cooper JP. 2010. Telomeric strategies: means to an end. Annu Rev Genet. 44:243–269. 21047259 10.1146/annurev-genet-102108-134841

[jkab360-B16] Keener R , Connelly CJ, Greider CW. 2019. Tel1 activation by the MRX complex is sufficient for telomere length regulation but not for the DNA damage response in *Saccharomyces cerevisiae*. Genetics. 213:1271–1288. 31645360 10.1534/genetics.119.302713 PMC6893380

[jkab360-B17] Lendvay TS , Morris DK, Sah J, Balasubramanian B, Lundblad V. 1996. Senescence mutants of *Saccharomyces cerevisiae* with a defect in telomere replication identify three additional *EST* genes. Genetics. 144:1399–1412. 8978029 10.1093/genetics/144.4.1399 PMC1207693

[jkab360-B18] Lin JJ , Zakian VA. 1996. The *Saccharomyces CDC13* protein is a single-strand TG_1-3_ telomeric DNA-binding protein in vitro that affects telomere behavior *in vivo*. Proc Natl Acad Sci U S A. 93:13760–13765. 8943008 10.1073/pnas.93.24.13760 PMC19417

[jkab360-B19] Lingner J , Hughes TR, Shevchenko A, Mann M, Lundblad V, et al 1997. Reverse transcriptase motifs in the catalytic subunit of telomerase. Science. 276:561–567. 9110970 10.1126/science.276.5312.561

[jkab360-B20] Liu J , Hong X, Wang L, Liang CY, Liu JP. 2021. Sir4 deficiency reverses cell senescence by sub-telomere recombination. Cells. 10:778. 33915984 10.3390/cells10040778 PMC8066019

[jkab360-B21] Lundblad V , Szostak JW. 1989. A mutant with a defect in telomere elongation leads to senescence in yeast. Cell. 57:633–643. 2655926 10.1016/0092-8674(89)90132-3

[jkab360-B22] Moretti P , Freeman K, Coodly L, Shore D. 1994. Evidence that a complex of SIR proteins interacts with the silencer and telomere-binding protein RAP1. Genes Dev. 8:2257–2269. 7958893 10.1101/gad.8.19.2257

[jkab360-B23] Nugent CI , Hughes TR, Lue NF, Lundblad V. 1996. Cdc13p: a single-strand telomeric DNA-binding protein with a dual role in yeast telomere maintenance. Science. 274:249–252. 8824190 10.1126/science.274.5285.249

[jkab360-B24] Pennock E , Buckley K, Lundblad V. 2001. Cdc13 delivers separate complexes to the telomere for end protection and replication. Cell. 104:387–396. 11239396 10.1016/s0092-8674(01)00226-4

[jkab360-B25] Peterson SE , Stellwagen AE, Diede SJ, Singer MS, Haimberger ZW, et al 2001. The function of a stem-loop in telomerase RNA is linked to the DNA repair protein Ku. Nat Genet. 27:64–67. 11138000 10.1038/83778

[jkab360-B26] Pfingsten JS , Goodrich KJ, Taabazuing C, Ouenzar F, Chartrand P, et al 2012. Mutually exclusive binding of telomerase RNA and DNA by Ku alters telomerase recruitment model. Cell. 148:922–932. 22365814 10.1016/j.cell.2012.01.033 PMC3327133

[jkab360-B27] Ritchie KB , Mallory JC, Petes TD. 1999. Interactions of *TLC1* (which encodes the RNA subunit of telomerase), *TEL1*, and *MEC1* in regulating telomere length in the yeast *Saccharomyces cerevisiae*. Mol Cell Biol. 19:6065–6075. 10454554 10.1128/mcb.19.9.6065 PMC84515

[jkab360-B28] Roy R , Meier B, McAinsh AD, Feldmann HM, Jackson SP. 2004. Separation-of-function mutants of yeast Ku80 reveal a Yku80p-Sir4p interaction involved in telomeric silencing. J Biol Chem. 279:86–94. 14551211 10.1074/jbc.M306841200

[jkab360-B29] Schulz VP , Zakian VA. 1994. The Saccharomyces *PIF1* DNA helicase inhibits telomere elongation and de novo telomere formation. Cell. 76:145–155. 8287473 10.1016/0092-8674(94)90179-1

[jkab360-B30] Shampay J , Szostak JW, Blackburn EH. 1984. DNA sequences of telomeres maintained in yeast. Nature. 310:154–157. 6330571 10.1038/310154a0

[jkab360-B31] Sherman F. 2002. Getting started with yeast. Methods Enzymol. 350:3–41. 12073320 10.1016/s0076-6879(02)50954-x

[jkab360-B32] Sholes SL , Karimian K, Gershman A, Kelly TJ, Timp W, et al 2021*.* Chromosome specific telomere lengths and the minimal functional telomere revealed by nanopore sequencing. bioRxiv. doi: 10.1101/2021.06.07.447263. 10.1101/gr.275868.121 PMC8997346 34702734

[jkab360-B33] Singer MS , Gottschling DE. 1994. *TLC1*: template RNA component of *Saccharomyces cerevisiae* telomerase. Science. 266:404–409. 7545955 10.1126/science.7545955

[jkab360-B34] Stellwagen AE , Haimberger ZW, Veatch JR, Gottschling DE. 2003. Ku interacts with telomerase RNA to promote telomere addition at native and broken chromosome ends. Genes Dev. 17:2384–2395. 12975323 10.1101/gad.1125903 PMC218076

[jkab360-B35] Strecker J , Stinus S, Caballero MP, Szilard RK, Chang M, et al 2017. A sharp Pif1-dependent threshold separates DNA double-strand breaks from critically short telomeres. eLife. 6:e23783. 28826474 10.7554/eLife.23783 PMC5595431

[jkab360-B36] van Mourik PM , de Jong J, Agpalo D, Claussin C, Rothstein R, et al 2016. Recombination-mediated telomere maintenance in *Saccharomyces cerevisiae* is not dependent on the Shu complex. PLoS One. 11:e0151314. 26974669 10.1371/journal.pone.0151314 PMC4790948

[jkab360-B37] van Mourik PM , de Jong J, Sharma S, Kavsek A, Chabes A, et al 2018. Upregulation of dNTP levels after telomerase inactivation influences telomerase-independent telomere maintenance pathway choice in *Saccharomyces cerevisiae*. G3 (Bethesda). 8:2551–2558. 29848621 10.1534/g3.118.200280 PMC6071591

[jkab360-B38] Wellinger RJ , Zakian VA. 2012. Everything you ever wanted to know about *Saccharomyces cerevisiae* telomeres: beginning to end. Genetics. 191:1073–1105. 22879408 10.1534/genetics.111.137851 PMC3415994

[jkab360-B39] Wotton D , Shore D. 1997. A novel Rap1p-interacting factor, Rif2p, cooperates with Rif1p to regulate telomere length in *Saccharomyces cerevisiae*. Genes Dev. 11:748–760. 9087429 10.1101/gad.11.6.748

[jkab360-B40] Wu Y , Zakian VA. 2011. The telomeric Cdc13 protein interacts directly with the telomerase subunit Est1 to bring it to telomeric DNA ends in vitro. Proc Natl Acad Sci U S A. 108:20362–20369. 21969561 10.1073/pnas.1100281108 PMC3251085

[jkab360-B41] Zappulla DC , Goodrich KJ, Arthur JR, Gurski LA, Denham EM, et al 2011. Ku can contribute to telomere lengthening in yeast at multiple positions in the telomerase RNP. RNA. 17:298–311. 21177376 10.1261/rna.2483611 PMC3022279

